# Containerless Bioorganic Reactions in a Floating Droplet by Levitation Technique Using an Ultrasonic Wave

**DOI:** 10.1002/advs.202002780

**Published:** 2020-12-16

**Authors:** Teruhiko Matsubara, Kenjiro Takemura

**Affiliations:** ^1^ Department of Biosciences and Informatics Faculty of Science and Technology Keio University 3‐14‐1 Hiyoshi, Kohoku‐ku Yokohama Kanagawa 223‐8522 Japan; ^2^ Department of Mechanical Engineering Faculty of Science and Technology Keio University 3‐14‐1 Hiyoshi, Kohoku‐ku Yokohama Kanagawa 223‐8522 Japan

**Keywords:** acoustic levitation, click chemistry, containerless, enzymatic reaction, radical polymerization

## Abstract

To ensure sustainable consumption and production patterns, alternative process design without plastics for chemical and biological reactions will benefit future generations. Reaction flasks used in chemical and biological laboratories have been made from glass, metals, and plastics so far. If containerless processing can be realized, researchers will have a next‐generation reaction process, which will be reactor and plastic‐free, and without risks of unforeseen issues induced by contact with reactions flasks, including contamination and alteration of the reactants. Here, polymerization, click chemistry, and enzymatic reactions can proceed effectively in a floating droplet at a node of standing wave generated by ultrasonic levitation. These results demonstrate that floating droplets levitated by acoustic waves can represent a revolutionary containerless reactor for performing various reactions in the fields of chemistry and biology.

## Graphical abstract

This work provides exciting possibilities of a containerless, plastic‐free biochemical reactor. A droplet can be floated in the air at a node of a standing wave generated by ultrasonic levitation. Various chemical and bioorganic reactions, such as polymerization, click chemistry, and enzymatic reactions, are found to effectively proceed in the levitated droplet.

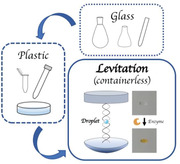

Although chemical and biological reactions in laboratories have been performed in glass and metals devices/containers during the past few hundred years, polymeric materials like polystyrene and polypropylene are being commonly used for biological experiments. Plastics are lightweight, cheap, and disposable; however, the weight of the plastic wastes, such as of pipette tips, tubes, and dishes, generated in biological laboratories is estimated to be ≈60 kg per person, in a year.^[^ ^1^ ^]^ Microplastics, which are small particles of plastic material with the size of 5 mm or smaller, including nanoplastics, have emerged as a problem recently.^[^ ^2^ ^]^ Microplastics are generated from pieces of plastic products, and can have a negative impact on animal and/or human health through biological accumulation.^[^ ^3^ ^]^ Many people are involved in a plastic‐free movement to reduce plastic pollution in the environment by choosing to refuse single‐use plastics in commercial industries and home use, but the elimination of single‐use plastics through reduction or reuse has not become widespread among scientists.^[^ ^1^ ^]^

Regulation of adsorption of compounds and biomolecules at an air–water–solid interface can affect the outcome of experiments in the fields of chemistry and biology.^[^ ^4^ ^]^ For example, plastics can easily adsorb proteins, and coating with bovine serum albumin and 2‐methacryloyloxyethyl phosphorylcholine polymer is often performed to minimize nonspecific background.^[^ ^5^, ^6^ ^]^ Recently, Matsubara et al. reported the ultrasensitive detection of human and avian influenza virus using a boron‐doped diamond electrode that has unique characteristic of weak molecule adsorption.^[^ ^7^, ^8^ ^]^ If a reaction solution is able to have no contact with the reactor material, target molecules would not be influenced by adsorption onto the surface of the reaction flask. Nearly two decades ago, floating of objects such as polymers and heavy tungsten spheres by acoustic levitation was reported.^[^ ^9^, ^10^ ^]^ If a water droplet is stably floated in air, a useful air–water interface may be available for reaction vessels of organic syntheses as well as gold particle assembly.^[^ ^11^ ^]^ However, to our knowledge, organic synthesis and bioorganic reactions in such droplets have not been reported so far. In the present study, we show that a levitated floating droplet has considerable potential as a reactor for chemical and biological reactions.

We prepared a single‐axis acoustic levitator that consists of an ultrasonic transducer device with a concave horn and a reflector (**Figure** **1**a). To obtain an acoustic field, an ultrasonic wave with 60 kHz frequency at maximum intensity of 1.6 W cm^−2^ was generated from bottom up of the horn along with a gravitational direction. The wave is reflected with the reflector, and a standing wave was generated by manually adjusting a distance between the horn and the reflector. A water droplet in the range of 1 to 10 µL could be fixed at a node of the standing wave, and two or more droplets were levitated on multiple nodes (Figure S1a,b, Supporting Information). The working frequency of the transducer was 60 kHz, and the distance between the nodes was calculated from the equation, *λ* = *c*/*f*, where *λ*, *c*, and *f* are the wavelength (m), speed of sound in air (340 m s^−1^), and frequency (Hz), respectively. From this equation, *λ* was calculated to be 5.67 mm. As we estimated, the observed distance of ≈3 mm between the droplets corresponded to the distance between the nodes (half wavelength, 2.8 mm). To clarify the liquid levitation, the droplet was colored with gel loading dye (0.01% bromophenol blue) and was absorbed on a cotton swab (Figure 1b and Movie S1, Supporting Information).

**Figure 1:**
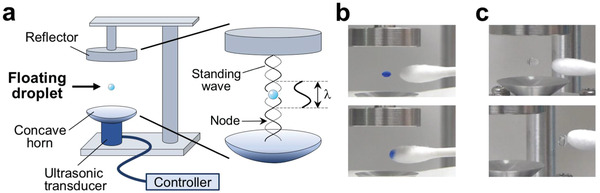
A floating droplet generated by ultrasonic levitation in a standing wave. a) Schematic representation of a single‐axis acoustic levitator. Ultrasonic transducer, 60 kHz. Concave horn, 20 mm in diameter. Standing wave is generated between transducer and reflector, and the distance of each nodes was calculated to be 2.8 mm, which is half of the wavelength. b) Absorption of colored droplet with gel loading dye (0.01% bromophenol blue) on a cotton swab (see Movie S1, Supporting Information). c) Polymerization of acrylamide in a floating droplet. Levitated polyacrylamide gel (upper) was collected using the surface of a cotton swab (lower). Shape of the polyacrylamide gel was still retained after attachment to the cotton swab (see Movie S2, Supporting Information).

Polyacrylamide gel electrophoresis is commonly used to separate proteins and nucleic acids based on the molecular weight.^[^ ^12^ ^]^ This gel is obtained by polymerization of acrylamide and bis‐acrylamide (N,N′‐methylenebisacrylamide) in water by radical polymerization (Figure S2a, Supporting Information). A mixed solution of acrylamide and bis‐acrylamide was levitated, and then initiator and catalyst were added to the droplet to initiate the polymerization (15 s in Movie S2, Supporting Information). The droplet turned into a gel within 1 min, and the gel was then collected with a cotton swab to demonstrate gelation of the solution visually (Figure 1c).

Bioorthogonal reactions for conjugation of functional modules to target biomolecules are used to investigate their functions in vitro and in vivo. Especially, the Cu(I)‐catalyzed Huisgen 1,3‐dipolar cycloadditions of azides with alkynes, called the “click reaction,” is often performed in the field of chemical biology.^[^ ^13^, ^14^ ^]^ This reaction proceeds in water rather than in an organic solvent; hence, this reaction can be used for the modification of water‐soluble biomolecules with functional groups.^[^ ^15^ ^]^ Here, Fmoc‐azidolysine (compound **2**), wherein the *ε*‐amino group of lysine side chain is replaced by an azide, was conjugated with an alkyne‐modified biotin (**1**) in a reaction tube to give a product (**3**) containing a 1,4‐disubstituted‐1,2,3‐triazolyl moiety (**Figure** **2**a). Concurrently, a solution containing (**2**) (1 equivalent), alkyne‐modified biotin **1** (1 equivalent), ascorbic acid (reduction reagent, 0.5 equivalent), tris(3‐hydroxypropyltriazolylmethyl)amine (THPTA) (copper stabilizing ligand, 5 equivalent) was levitated, followed by the addition of CuSO_4_ (catalyst, 0.25 equivalent). The progress of the reactions was followed using high‐performance liquid chromatography (HPLC) by monitoring the appearance of product **3** and an accompanying decrease in (**2**) (Figure 2b). Based on the electrospray ionization (ESI) mass spectrum, the peak in retention time at 29 min was assigned to product **3** as the [M+H]^+^ ion (*m*/*z* 852.4). The peak of the azide in the tube finally disappeared at 20 min (Figure S2b, Supporting Information), while the reaction in the droplet was completed within 5 min (95 ± 3% yield in 1 min) (Figure 2c). Thus, the click reaction in a floating droplet proceeded at least four times faster than that in a tube, indicating that the floating technology has great potential to enable the realization of a containerless synthetic chemical reactor.

**Figure 2:**
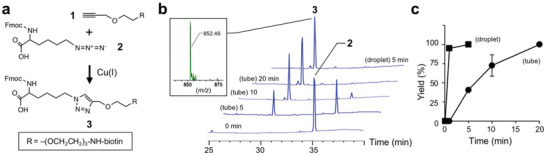
Chemical synthesis in the floating droplet. a) Cu(I)‐catalyzed click reaction. Conjugation of alkyne (Biotin‐PEG_4_‐alkyne, **1**) with azide (Fmoc‐azidolysine, **2**) to give a product **3**. b) HPLC charts of the time‐course for click reaction in a tube (5–20 min) or in the levitated droplet (5 min). Fmoc‐containing compounds **2** and **3** were detected at 220 nm. The product **3** was characterized by ESI mass spectrometry (inset). c) Summary of the time‐course for the click reaction in the tube or in the levitated droplet. The reaction yield (%) was plotted against reaction time. Data are average values ± standard deviations (*n* = 3).

We next designed bioorganic reactions in the droplet using enzymes. We first chose a visually perceivable enzymatic reaction with the colorimetric substrate *o*‐phenylenediamine (OPD). OPD is oxidized by peroxidase in the presence of hydrogen peroxide (H_2_O_2_), leading to a reaction product that is orange‐brown in color.^[^ ^16^ ^]^ The horseradish peroxidase (HRP) is one of the most used enzymes for biological assays such as enzyme‐linked immunosorbent assay (ELISA), Western blotting, and various immunohistochemical assays. A mixed solution containing OPD and peroxidase‐conjugated avidin was levitated, and H_2_O_2_ was added to the droplet to start the enzymatic reaction. The color of the levitated droplet gradually deepened with time, and the enzymatic reaction was confirmed based on the continuous color changes during 15 min (**Figure** **3**a). The volume of the collected OPD solution was reduced to about 6 µL due to evaporation of water (Figure S2c, Supporting Information). Typically, the oxidation reaction takes 10–20 min in ELISA, indicating that the enzymatic reaction in the floating droplet is comparable and might be useful in biological studies.

**Figure 3:**
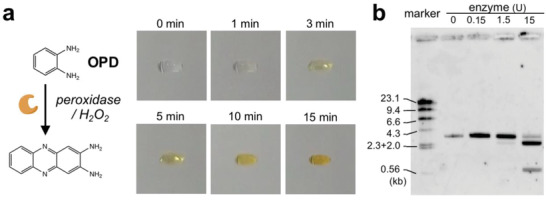
Bioorganic reactions in the floating droplet. a) Enzymatic oxidation of the colorimetric substrate by peroxidase. Color of *o*‐phenylenediamine (OPD) was developed after 15 min of reaction with peroxidase in the presence of hydrogen peroxide. b) DNA digestion by restriction enzyme in the levitated droplet. A linear DNA fragment (3.4 kb, 0.2 µg) was digested using HindIII (0.15, 1.5, and 15 U) for 15 min to give rise to two fragments (2.6 and 0.8 kb). The reaction mixture was analyzed by 0.6% agarose gel electrophoresis to determine the size of each product. Marker, *λ*/HindIII digestion (0.25 µg).

DNA manipulations, such as ligation, digestion, and amplification, by enzymes are required for genetic engineering. In the present study, enzymatic digestion of a linearized plasmid DNA was performed. The linear DNA fragment (3.4 kb, 200 ng) was incubated with a restriction enzyme (0.15–15U), and the digested short fragments of 2.6 and 0.8 kb were identified by agarose gel electrophoresis (Figure 3b). In addition, full length DNA was found to be retained after levitation, indicating that marked damage to the DNA fragment was not observed in the present condition (Figure S2d, Supporting Information). Damages to single‐strand DNA by ultrasonic exposure of cultured cells to 1.6 MHz frequency at a continuous 8 W cm^−2^ spatial peak temporal average intensity for 10 min have been reported by Miller.^[^ ^17^ ^]^ This damage is considered to be induced by cavitation that generates reactive oxygen species (ROS) and physical stress.^[^ ^18^ ^]^ However, if the intensity is less than 2 W cm^−2^, no damages to macromolecules such as DNA are observed, although ROS is generated.^[^ ^19^ ^]^ Indeed, ultrasonic exposure (within 20 min) in the present study was performed at an intensity of less than 1.6 W cm^−2^, resulting in no damage to the DNA. In addition, 99.9% of the sound energy is reflected at the water–air interface due to the difference in acoustic impedance between water and air.^[^ ^20^ ^]^ Diagnostic and therapeutic ultrasound imaging with a frequency of 2–40 MHz have been widely used in clinical cases. Since the scattered power is proportional to the fourth power of the frequency of the wave, the frequency of 60 kHz used in this study is far from the frequency required to cause ROS‐mediated damages.^[^ ^20^, ^21^ ^]^ These results support that DNA and other biomacromolecules are treatable in the floating droplets.

In combination with the existing technology, this levitation system has a significant potential in industrial applications. The droplet dynamics have been investigated; for example, the shape of the droplet levitated can be manipulated by adjusting the sound intensity or field distribution.^[^ ^22^ ^]^ In the present study, we simply levitated the droplet; however, the acoustic manipulations should provide not only the control of chemical and bioorganic reactions at the gas–liquid interface but also fabrication of soft materials with unique morphologies. Using the present system, the volume of the droplet that we can treat is only 1–20 µL; however, this volume range is sufficient to perform most biochemical experiments such as enzymatic reactions. If we need to treat a large volume (in mL and more), dozens or hundreds of droplets could be generated using combined mechanical instruments automatically.^[^ ^23^ ^]^ Furthermore, as the size of the set of the transducer and refractor is 5 cm in diameter and less than 15 cm high, the reaction in the levitated droplet can be easily performed at a certain temperature, humidity, and atmosphere in a closed box. Since the weight of the present system is ≈1 kg, it is easy portable; therefore, this can be used under various situations on the earth as well as at the space station.

In conclusion, we found that organic synthesis, polymerization, and enzymatic reactions can proceed in levitated droplets. Although mechanical stress and ROS generated from cavitation under ultrasonication with the high frequency (>40 MHz) may damage the target materials in the levitated droplets, the frequency used in this study was within the MHz range, indicating the scattered powers is safety level. This levitation system would facilitate a containerless reaction on earth as well as under microgravity conditions such as on the space station. We thus propose this system as a next‐generation reaction process.

## Experimental Section

##### Single‐Axis Ultrasonic Levitator

The single‐axis acoustic levitator is composed of a reflector and a Langevin type ultrasonic transducer device (frequency, 60 kHz; concave horn, 20 mm diameter; intensity, <1.6 W cm^−2^; length, 39 mm; Honda Electronics Co., Ltd., Japan).^[^ ^10^ ^]^ The distance between the reflector and the bottom of the horn was ≈15 mm. As the strongest acoustic forces are generated by standing sound waves, the fine adjustment of the distance was made manually to cause resonance.^[^ ^24^ ^]^ An airborne acoustic wave at this frequency has a wavelength of 5.67 mm at 25 °C, which allows the levitation of objects with the distance of 2.8 mm (half‐wavelength).

##### Polymerization of Acrylamide

Polyacrylamide gels were prepared by radical polymerization of acrylamide and bis‐acrylamide (N,N′‐methylenebisacrylamide).^[^ ^25^ ^]^ Polymerization was initiated by ammonium peroxydisulfate (APS) in the presence of N,N,N′,N′‐tetramethylethylenediamine (TEMED; catalyst). Briefly, a mixed solution of 3.2 µL of 30% acrylamide (14.5 g of acrylamide and 0.5 g of bis‐acrylamide in 50 mL of water), 2.4 µL of 5× Tris‐borate‐ethylenediaminetetraacetic acid (EDTA) buffer (5× TBE), and 3.2 µL water was levitated. A 1:1 mixed solution (1.2 µL) of 10% TEMED and 10% APS was prepared, and then immediately added to the floating droplet (total 10 µL). The droplet turned into a gel within 1 min, and the gel was collected with a cotton swab.

##### Click Reaction

A Cu(I)‐catalyzed Huisgen 1,3‐dipolar cycloaddition (click reaction) of azides with alkynes was performed as described previously with minor modifications.^[^ ^26^ ^]^ A mixed solution of *N* ^*α*^‐Fmoc‐*ε*‐azido‐l‐norleucine **2** (Fmoc‐azidolysine, Merck) (1.0 µL of 10 × 10^−3^ m solution in N,N‐dimethylformamide (DMF), 10 nmol, 1 equivalent), alkyne‐modified biotin **1** (Biotin‐PEG_4_‐alkyne, Sigma‐Aldrich) (1.0 µL of 10 × 10^−3^ m solution in 50% methanol, 10 nmol, 1 equivalent), sodium ascorbate (1.0 µL of 50 × 10^−3^ m solution in 50% methanol, 5 nmol, 0.5 equivalent), and tris(3‐hydroxypropyltriazolylmethyl)amine (THPTA) (0.5 µL of 100 × 10^−3^ m solution in water, 50 nmol, 5 equivalent) was prepared. After levitation of the mixed solution, 0.5 µL CuSO_4_ (50 × 10^−3^ m, 2.5 nmol, 0.25 equivalent) was added to start click reaction. The droplet was collected after 1, 3, 5, 10, and 20 min. To stop the reaction, the collected solution was diluted five times with water, an excess (10 equivalent) of EDTA relative to Cu was added, and then frozen until analysis using reversed‐phase HPLC.^[^ ^27^ ^]^ In the tube experiments, the click reaction was performed in 0.5 mL polypropylene tubes (Eppendorf, Hamburg, Germany).

The progress of the reaction was followed using HPLC by monitoring the appearance of product (**3**) (Fmoc‐lysine‐conjugated biotin), with an accompanying decrease in Fmoc‐azidolysine (**2**). The analyses were performed on ODS column (250 mm × 4.6 mm I.D., detection at 220 nm) with a linear gradient of water containing 0.1% TFA and acetonitrile containing 0.1% TFA at a flow rate of 1 mL min^−1^. Fmoc‐containing compounds of (**2**) and (**3**) were detected at 220 nm. The retention time of the product (**3**) and the azide (**2**) was ≈29 and 35 min, respectively. The product (**3**) (calculated [M+H]^+^, *m*/*z* 852.4) was identified by an ESI/ion trap mass spectrometry (amaZon SL, Bruker Daltonics).

##### Color Development of OPD

OPD dihydrochloride is often used as a substrate in enzymatic reactions with horseradish peroxidase in ELISA.^[^ ^28^ ^]^ A 5 mg OPD tablet (Sigma‐Aldrich) was dissolved in 12.5 mL citric acid‐phosphate buffer at pH 5 (0.4 mg OPD mL^−1^; OPD solution). HRP‐conjugated streptavidin (Sigma‐Aldrich) was dissolved in phosphate‐buffered saline at pH 7.4 (PBS) (0.01 mg mL^−1^; HRP solution). OPD solution (23 µL) was mixed with HRP solution (0.5 µL). A portion (10 µL) of the mixed solution was levitated, and 0.8 µL of 0.3% H_2_O_2_ was added (0 min). After 15 min, the solution changed color to orange‐brown, and a 44% decrease in its volume was observed (6.0 µL final volume).

##### Plasmid DNA Digestion by Restriction Enzyme

Prior to use for levitation, a linear DNA fragment was prepared by digestion of pAcGFP1 vector (3.4 kb, Clontech) with EcoRI. The solution (9 µL) of the linear DNA fragment (0.2 µg) in buffer was levitated, and 1 µL of HindIII (0.1, 1.5, and 15 U) was then added to the droplet. After 15 min of levitation, the reaction mixture was collected and stored on ice. 2 µL of the mixture (40 ng) were then analyzed by 0.6% agarose gel electrophoresis in 0.5× Tris‐acetate EDTA buffer (100 V, 30 min). DNA fragment was visualized by ethidium bromide.

##### Statistical Analysis

All experiments were performed in triplicate and repeated at least twice with similar results. For summarizing of HPLC analyses (Figure 2c), data shown are the average values ± standard deviations of three independent experiments.

## Conflict of Interest

The authors declare no conflict of interest.

## Supporting information

Supporting Information

Supplemental Movie 1

Supplemental Movie 2
