# Supplementary material for: Containerless Bioorganic Reactions in a Floating Droplet by Levitation Technique Using an Ultrasonic Wave
Source: Adv Sci (Weinh). 2020 Dec 16;8(3):2002780. doi: 10.1002/advs.202002780 (PMC7856899; doi:10.1002/advs.202002780)
Supplement: Supplementary file 1 — Supporting Information [file ADVS-8-2002780-s001.pdf]

WILEY-VCH

© 2020 Wiley-VCH GmbH

ADVANCED SCIENCE
Open Access

Supporting Information

for *Adv. Sci.*, DOI: 10.1002/advs.202002780

Containerless Bioorganic Reactions in a Floating Droplet by Levitation Technique Using an Ultrasonic Wave

Teruhiko Matsubara, and Kenjiro Takemura*

Supporting Information

Containerless bioorganic reactions in a floating droplet by levitation technique using an ultrasonic wave

Teruhiko Matsubara, Kenjiro Takemur*a

Contents

Figure S1. Robustness of water droplets generated by ultrasonic levitation.

Figure S2. Chemical and bioorganic reactions in tube and levitated droplet.

Movie S1. A floating droplet (the former 5 s) and absorption of a colored droplet with gel loading dye on a cotton swab (the latter 5 s) (total 10 s).

Movie S2. Polymerization of acrylamide in a floating droplet (1 min 30 s).

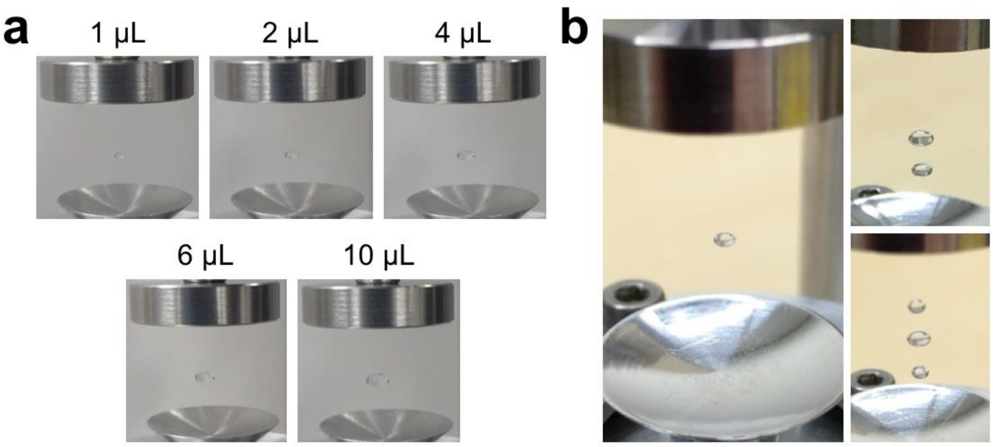

Figure S1. Robustness of water droplets generated by ultrasonic levitation.

(a) Stable levitation was observed independent of the volumes in the range of 1 to 10 µL. (b) Ultrasonic levitation of water droplets (10 µL) in air. Two (5 and 10 µL, *upper right*) and three (two 5 µL and 10 µL, *lower right*) levitated droplets are shown, and the distance (approximately 3 mm) of the drops corresponds to half that of the wavelength (λ, 2.8 mm).

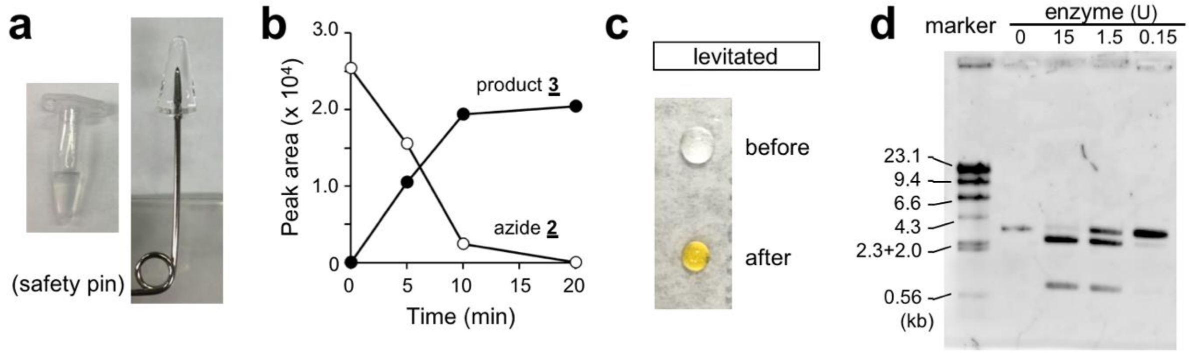

Figure S2. Chemical and bioorganic reactions in tube and levitated droplet.

(a) Polyacrylamide gel after polymerization in a 0.5-mL tube (130 µL). (b) Typical time-course for the click reaction in the tube. The peak area of the HPLC chart was plotted against reaction time. (c) OPD solution before and after enzymatic oxidation by peroxidase in the levitated droplet (10 µL). (d) DNA digestion (3.4 kb, 0.2 µg) by HindIII (0.15, 1.5, and 15 U) for 15 min in a tube. Two fragments (2.6 kb and 0.8 kb) were observed after digestion, followed by 0.6% agarose gel electrophoresis in 0.5× Tris-acetate EDTA buffer (100 V, 30 min). Marker, λ/HindIII digestion (0.25 µg).
